# Strategies for achieving viral hepatitis C micro-elimination in the Netherlands

**DOI:** 10.1186/s41124-018-0040-9

**Published:** 2018-09-29

**Authors:** P. A. M. Kracht, J. E. Arends, K. J. van Erpecum, A. Urbanus, J. A. Willemse, A. I. M. Hoepelman, E. A. Croes

**Affiliations:** 1 Department of Internal medicine and Infectious disease, Utrecht University, University Medical Center Utrecht, Utrecht, the Netherlands; 2 Department of Gastroenterology and Hepatology, Utrecht University, University Medical Center Utrecht, Utrecht, the Netherlands; 3 Centre for Infectious Disease Control, National Institute for Public Health and the Environment, Bilthoven, The Netherlands; 4 Dutch Liver Patient Association (NLV), Hoogland, the Netherlands; 5 Netherlands Institute of Mental Health and Addiction (Trimbos Institute), Utrecht, the Netherlands

**Keywords:** Hepatitis C virus, Micro-elimination, Hep-CORE, HCV cascade of care

## Abstract

The Netherlands is striving to achieve national elimination of the hepatitis C virus (HCV) as one of the first countries worldwide. The favorable HCV epidemiology with both low prevalence and incidence, together with access to care and treatment, present excellent conditions to further build on towards this objective. The Dutch national plan on viral hepatitis, introduced in 2016, defines targets in the HCV healthcare cascade and provides a structural framework for the development of elimination activities. Since many different stakeholders are involved in HCV care in the Netherlands, focus has been placed on micro-elimination initiatives as a pragmatic and efficient approach. These numerous micro-eliminations projects have brought the Netherlands closer to HCV elimination. In the near future, efforts specifically have to be made in order to optimize case-finding strategies and to successfully accomplish the nationwide implementation of the registration and monitoring system of viral hepatitis mono-infections, before this final goal can be reached. The upcoming years will then elucidate if the Dutch’ hands on approach has resulted in sufficient progress against HCV and if the Netherlands will lead the way towards nationwide HCV elimination.

## Background

Global elimination and eradication of the hepatitis C virus (HCV) has become the ultimate endeavor and final objective ever since the introduction of highly effective direct-acting antivirals (DAAs). The once fatal disease has thus been transformed into an infection that can effortlessly be cured, provided that one has access to care and therapy. Consequently, the World Health Organization (WHO) even envisioned universal elimination of the HCV to be accomplished by the year 2030 [1]. To this end, the WHO urged countries to develop and implement national policies on viral hepatitis. However, the European Liver Patients Association (ELPA) Hep-CORE study revealed that 14/27 countries still did not have a written national plan for the management of hepatitis C and / or hepatitis B (HBV) in 2016. The Netherlands therefore subsequently introduced a national plan on viral hepatitis in 2016 defining targets for each step in the Dutch HCV healthcare cascade: 1) awareness and prevention; 2) testing and diagnosis; 3) linkage to care; 4) access to medication and 5) monitoring and evaluation, in order to eventually achieve HCV elimination [2]. National coordination of HCV elimination however is complicated by (regional) differences in HCV patient subpopulations and the high number of stakeholders involved in HCV care. Considering these diversities, a uniform nationwide strategy targeting all HCV patients is unlikely to be the key solution to finally eliminate HCV. A more pragmatic approach would be to apply more focus and work towards ‘micro-elimination’ in different HCV subpopulations (e.g., individuals with HIV or hemophilia, (ever) injection drug users, migrants from high endemic countries, health care workers, prisoners). Micro-elimination, by way of targeting smaller and clearly delineated HCV risk groups, allows for faster and more efficient delivery of interventions. For this reason, micro-elimination as a ‘bottom-up’ approach may be a more feasible and efficient path to nationwide HCV elimination [3]. Various stakeholders involved in HCV care have devoted their efforts to micro-elimination initiatives in the Netherlands, including: a) awareness campaigns directed at (ever) injection drug users and migrants but also health care workers; b) screening strategies in risk-groups to find undiagnosed persons with HCV, as recommended by the national Health Council [4]; c) regional and nationwide retrieval projects of lost to follow-up previously diagnosed patients; d) HCV healthcare pathways in addiction clinics and primary care to promote and guide linkage to care; e) registry and close monitoring of HCV subpopulations (registry of HIV/HCV coinfected managed by the Dutch HIV monitoring foundation and the hemophilia treatment center follows all HCV patients with hemophilia). However, the success of all (micro-) elimination efforts hinges on solid epidemiologic data on HCV prevalence and incidence in all HCV risk groups. Adequate registration and monitoring is an imperative element of HCV elimination and achieving this may be one of the most difficult obstacles to overcome in the elimination process.

This paper will describe (trends in) HCV epidemiology in the Netherlands, the micro-elimination progress in different HCV risk groups and also outline the strategies that have been employed by different stakeholders to improve uptake and retention in each step of the Dutch HCV healthcare cascade. Finally, we will elaborate on the Dutch progress towards nationwide HCV elimination and elucidate if the Netherlands may be among the first countries to achieve this final ambition in the near future.

## Hepatitis C prevalence and incidence in the Netherlands

Although it is generally accepted that the Netherlands is a low prevalence region for HCV, only few studies have ever been performed to ascertain its prevalence in the general population. Two cross-sectional serosurveys in 1996 and 2007 calculated a national prevalence of 0.1% and 0.3%, respectively [5, 6]. Of note, groups from HCV endemic regions were considered underrepresented in the ‘96 survey and therefore a higher proportion (70%) of non-Dutch nationalities were included in the ‘07 survey. In 2012, Vriend et al. applied a different approach (i.e. the Workbook method) to estimate HCV prevalence in the Netherlands. The Workbook method incorporates lowest and highest available risk group based prevalence estimates which are subsequently multiplied by the different population sizes to generate an absolute number of HCV infected individuals per risk group. This study reported an estimated HCV seroprevalence of 0.22% which had been averaged from the lowest and highest total estimate of 0.07% and 0.37% respectively [7]. A recent update of this estimate, with more extensively defined prevalence estimates in different migrant populations, reported a 0.16% HCV seroprevalence in the Netherlands in 2017, which is still very similar to the older estimates from ‘96 and ‘07. This would correspond to 23,000 anti-HCV positive individuals in the Netherlands [8]. From a global perspective, it shows that the aforementioned HCV prevalence rate of 0.16% in the Netherlands is not only among the lowest in Western Europe but also one of the lowest worldwide [9–11]. Considering the HCV prevalence in distinct risk groups, the Dutch situation to some extent mimics the distribution in North America, Western Europe, and Australia, meaning that HCV infection is hyper endemic among people who (ever) inject(ed) drugs (PWID) (seroprevalence 39–74%) [12–16]. However, ongoing viral transmission of hepatitis C in the Dutch PWID group seems virtually non-existent: 44 cases of acute HCV were reported nationwide in 2016 of which < 5 were associated with intravenous drug use [17]. This is in line with the very low level of injecting among current drug users (estimated to be less than 1000 drug injectors in the whole country, with high access to clean injecting equipment). Nevertheless, underreporting due to an asymptomatic disease course cannot be ruled out. The low HCV incidence in PWID can also in part be explained by the decline in injection drug use (IDU) after the problematic 1960–1970’s as a consequence of overall diminished popularity but also due to the availability of physician-prescribed methadone treatment, needle exchange and other harm reduction programs [18, 19]. In 2015, the national number of recent injecting drug users was historically low (±500 i.e. 6% of all opiate users in addiction care) [20]. In the Netherlands, the first generation migrants emerge quantitatively as the major contributor to the Dutch national HCV burden (41–70%) [6–8]. However, the number of new infections due to immigration is not considered to have great impact on the total pool of chronically infected since the annual Dutch population growth as a cause of immigration is < 0.5% at the time of writing [21]. Also, the HCV prevalence in migrant populations in different nations has been described to be lower in comparison to the country of origin prevalence estimates [22]. The Netherlands, next to having a low prevalence, is also characterized by a low incidence of HCV infections that are mainly observed in the subgroup of HIV-infected persons (49 acute HCV infections in HIV-infected in 2016, i.e. 5.5/1000 person years, 95% CI 4.1–7.2). It was recently reported that the Dutch incidence rate of acute HCV in this group has decreased considerably, which is illustrative of an effective “treatment as prevention” approach [23]. However, current re-infection rates in HIV-infected (34/1000 person years in 2016) are still high and predominantly related to the involvement of men who have sex with men (MSM) in high-risk sexual activities (including ‘chemsex’, i.e. sex under the influence of psychoactive drugs) [24].

## Hepatitis C treatment effectiveness in different subpopulations

Various Dutch subpopulations with increased risk and/or prevalence of HCV can be identified as possible target groups for micro-elimination: the ‘low risk population’, migrants from high endemic countries, PWID, individuals with HIV, prisoners, MSM, patients with hemophilia, hemodialysis patients and health care workers (Table 1). As mentioned previously, to determine the success of HCV therapy uptake in subpopulations or otherwise assess the HCV micro-elimination progress, adequate patient monitoring systems are essential. In the Netherlands, several risk groups are closely monitored and (detailed) information on HCV therapy uptake is available in subpopulations such as HCV/HIV coinfected and HCV patients with an inherited bleeding disorder [24, 25]. With regard to the Dutch HIV population, annual screening for HCV infection is performed in those HIV positives in treatment with ongoing risk factors / behavior, irrespective of the presence of symptoms. Data from the Dutch HIV monitoring foundation (SHM), that includes 98% of all HIV-infected individuals who are retained in care, indicated that virtually all patients (*n* = 1439; 96.6%) had been screened at least once for HCV infection in 2017 [24]. Of note, the number of undiagnosed HIV/HCV coinfections is believed to be low (6% of the estimated 1750 undiagnosed Dutch HIV-infections) [24, 26]. Of those monitored HCV/HIV coinfected patients, 76% had been cured by February 2017 and from another 6% DAA therapy results were pending, totaling more than 80% clearance rate for this population [27]. Secondly, the van Creveldkliniek hemophilia treatment center (University Medical Center Utrecht, The Netherlands) reported that 27% of all 700 chronic HCV patients with an inherited bleeding disorder from a combined Dutch and UK cohort had been cured in April 2012 [25]. This proportion of cured patients, like in the HCV/HIV co-infected population, is expected to have increased dramatically since DAA-therapy became available.

**Table 1:** Hepatitis C micro-elimination progress in target populations in the Netherlands in 2017

	Population size (N)	HCV seroprevalence (%)	Total chronic HCV infections (HCV RNA (+)) (N)	HCV infections cured (N)/(%)	Source/Comments	Main actions/interventions to facilitate HCV elimination
HIV-infected	22,900	12%	1471 (R)	1124/76%	[24, 27]	• Behavioral counseling. • Once in a lifetime or frequent^c^ screening (depending on risk behavior).
Hemophilia patients (born < 1992)	NA	NA	700 (R)	190/27.1%	[25] (Combined Dutch & UK cohort)	• Once in a lifetime screening. • Treatment scale-up.
High-risk MSM (HIV-negative)^b^	NA	4,8%	NA	NA/NA	[57]	• Behavioral counseling. • Frequent^c^ screening • Early treatment in case of (re) infection.
Migrants from high endemic countries	1,527,032	NA	13,819 (E)	NA/NA	[58]	• Raise awareness of HCV through local/multimedia information campaigns. • Once in a lifetime screening for first-generation migrants with HCV prevalence ≥2% in country of origin.
PWID	14,000	39–74%	4040–7666 (C)	NA/NA	[7, 12–16, 59]	• Once in a lifetime or frequent^c^ screening (depending on risk behavior). • Treatment scale up.
Prisoners	10,194/each day	7.4–13.9%	558–1049 (C)	NA/NA	[60–62]	• Educate prison doctors on HCV. • Once in a lifetime or frequent^c^ screening (depending on risk behavior). • Include detainees in regular health insurance.
Hemodialysis patients	17,132	NA	NA	NA/NA	[63]	• Once in a lifetime screening.
Health care workers^d^	NA	NA	NA	NA/NA	–	• Once in a lifetime screening by employer.
General Dutch population	17,081,507^a^	0.1–0.4%	12,640–50,561 (C)	4427/8–35%	[7, 29]	• Raise awareness of HCV through multimedia information campaigns. • Educate general practitioners on HCV to increase compliance with viral hepatitis screening and referral guidelines. • Trace and treat HCV infected lost to follow-up.

*PWID* people who (have ever) inject(ed) drugs, *MSM* men who have sex with men, *NA* not available. (R) = reported numbers from publications of registries; (E) = estimated numbers reported in studies; (C) = calculated from seroprevalence estimates multiplied by .74 (assumed spontaneous clearance rate of 26%)

^a^ https://opendata.cbs.nl/statline/#/CBS/nl, Dutch population numbers in 2017

^b^ MSM with high-risk sexual activities

^c^ One to four times per year

^d^ Health care workers who perform hazardous tasks, putting them at risk for acquiring of transferring a hepatitis C infection (definition from the National Health Council) [4]

Also, the transmission of HCV to hemophilia patients due contaminated blood products has been halted with the introduction of anti-HCV testing in blood donors and of recombinant clotting factors [28]. Finally, data obtained from the Drug Information System of the National Health Care Institute (GIP) illustrate that between the years 2009 until the availability of DAAs in 2015 an estimated 4427 persons with HCV have been cured in the Netherlands (i.e. 19% of the ±23,000 estimated HCV antibody positive individuals) [29]. With regard to the remaining HCV risk groups, exact data is lacking and no reliable estimate of DAA therapy uptake in these subpopulations could be made. A HCV prevalence estimate was available in a number of groups (Table 1).

## Dutch hepatitis C healthcare cascade: Strategies towards micro-elimination

### Awareness and prevention

**Fig. 1:**
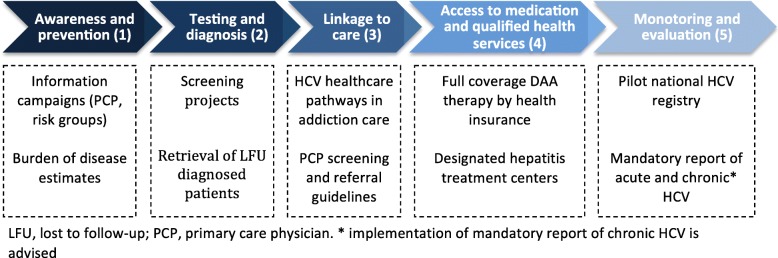
Dutch HCV care cascade

A summary of the main Dutch strategies towards HCV elimination is depicted in Fig. 1. In the Netherlands, several institutions involved in public healthcare are dedicated to increasing awareness and knowledge of HCV in risk groups and as well as in the general population. To this end, a large nation-wide multimedia HCV awareness campaign was implemented in 2009/2010, targeting individuals attending methadone clinics and also risk groups in the general population. In participating methadone clinics HCV test uptake was 62% during the campaign. At the end of the project, 257 additional HCV-carriers had been identified in methadone clinic attendees and the intervention was judged to be cost-effective in this group. In contrast, the intervention was not cost-effective in the general population although the number of anti-HCV tests in 25 laboratories had increased by 12.9% in comparison with previous years [30]. At the annual Dutch National Hepatitis Day in November 2017, another compendious media campaign with both radio and television broadcasts was launched by the Dutch Liver Patient Association (NLV) to raise awareness among patients, physicians but also health policy-makers for the increasing Dutch mortality rates as a cause of viral hepatitis [31]. This campaign has reached over ten million persons in the Netherlands (personal communication José Willemse, Executive director NLV).

### Testing and diagnosis: Screening and retrieval projects

A key factor in achieving HCV elimination lies in augmenting the case finding rate. Case finding can be further classified as: i) the identification of undiagnosed HCV patients (i.e. screening) and ii) tracing of previously diagnosed patients who are no longer in clinical care (i.e. retrieval). Since migrants have been shown to account for the majority of HCV infections in the Netherlands, several large screening projects in migrant groups from high endemic countries (a.o. Afghanistan, China, Egypt, Iraq, Turkey, Poland) have been performed. The yield of these projects however was low, despite the use of peers in some projects, and only a handful of viremic HCV (1–10 persons per project, i.e. 0.1–4.8%) were identified of which also a small number was already aware of their disease [32–38]. These low results could be explained by a ‘healthy volunteer’ effect which indicates that the participants of screening projects are in fact healthier than the index population [39]. Further initiatives are therefore still needed to encourage more individuals with a migration background to get tested for HCV. This is also supported by the Dutch National Health Council (HC) who as of November 2016 recommends HCV screening in all first generation migrant populations with a known HCV prevalence in the country of origin of ≥2%. At the moment however, screening projects targeting migrant populations at locations other than primary care practices, require approval from the Ministry of Health, Welfare and Sport (VWS) [4]. This prerequisite poses a barrier for renewed screening efforts in migrant subgroups (last project dates from 2013) in the present DAA-era in which therapeutic options have improved immensely. Consequently, HCV screening in first generation migrants currently falls under the responsibility of Primary Care Physicians (PCPs) in the Netherlands who are guided by their evidence-based professional ‘guideline on viral hepatitis’ from the Dutch College of General Practitioners (NHG) [40]. The NHG guideline in addition promotes screening in individuals with an increased ALT (≥1.5 times the upper limit of normal) in the general population who may have been at risk for HCV infection. However, due to the small number of hepatitis patients in each PCP practice and the limited knowledge on hepatitis, screening of hepatitis in PCP practices is suboptimal. Another subgroup with high HCV prevalence (Table 1) deserving enhanced screening efforts is the detention population. Although the Dutch Custodial Institution Agency introduced updated guidelines on HCV screening in 2016, promoting HCV screening upon confinement for those who have been at risk, non-compliance with those guidelines occurs regularly and the frequent relocation of detainees further complicates treatment initiation and adherence. In addition, the Ministry of Justice finances the Dutch prison healthcare system and reimbursing DAA-therapy poses a heavy burden on its total health care budget. This constitutes another barrier for testing and treating HCV-infected detainees.

Next to screenings projects, several retrieval projects of diagnosed patients who have been lost to follow-up have been executed in various regions in the Netherlands. Projects were initiated by different physicians involved in care for hepatitis patients: hospital based specialists (Gastroenterology, Infectious Diseases and Microbiology), PCPs, but also Public Health physicians. The most commonly applied method in those retrieval projects was the reevaluation of positive HCV diagnostics from the past 10–15 years from microbiological laboratories in order to identify untreated patients. Patients with presumed persistent HCV would then be reevaluated by their PCP and, if appropriate, invited back into clinical care. The proportion of chronic HCV patients who were lost to follow-up was reported to be as high as 38% [41–43]. Results of the main (published) Dutch retrieval projects are summarized in Fig. 3. The largest retrieval effort in the center of the Netherlands (REACH-project) has been the most successful thus far with 28.3% of all lost to follow-up patients traced [44]. One of the success factors may be that, in contrast to other endeavors, in the REACH-project patients were invited directly at the outpatient clinic without interference of the general practitioner. The REACH-project served as a pilot and subsequent nationwide roll-out of HCV retrieval is scheduled to start in 2018 (CELINE). The CELINE initiative constitutes a collaboration between hepatologists and Infectious diseases physicians of all eight Dutch Academic Medical Centers (HepNED) and aims to realize case ascertainment in > 50% those who previously tested positive for HCV in the next 3 years and also to include 95% of retrieved patients in a national registry [45].

### Linkage to care

The Dutch national plan on viral hepatitis underlined the importance of regional, multi-stakeholder, healthcare networks (i.e. hepatitis teams) in order to monitor and safeguard the local identification and linkage to care of HCV patients from all different risk group [2]. Such hepatitis teams have been installed successfully in various regions (Arnhem, Utrecht) and may serve as an example for other districts [32]. The “Breakthrough Project”, initiated by the Netherlands Institute of Mental Health and Addiction (Trimbos Institute) is another example of a collaborative between various stakeholders. The Trimbos Institute aspired to structurally improve the detection and linkage to care of HCV in Dutch addiction care clinics and to develop sustainable HCV referral cascades. To this end, two implementation projects based on the Breakthrough methodology (Breakthrough Project) were initiated between 2013 and 2016. Multiple multidisciplinary teams, including Gastroenterology specialists from a (nearby) hepatitis treatment center and nurses, medical doctors and managers from the local addiction care center, created a local and sustainable HCV referral pathway to secure linkage to care. Final results of the project are pending.

### Access to medication and qualified health services

In the Netherlands, virtually all patients have good access to healthcare and also, once diagnosed with HCV, to DAA therapy. As of November 2015, all available DAAs are reimbursed by basic health care insurance (which is obligatory in the Netherlands) irrespective of fibrosis stage, alcohol or drug use status. Basic healthcare insurance in the Netherlands however includes obligatory deductible excess, which added up to 385 euro per year in 2017 and 2018. This amount has to be paid by the policy holder before an insurance providers covers any expenses and may negatively affect one’s willingness to get tested for HCV, especially in individuals with lower socio-economic status. On average, the obligatory deductible excess is fully spend by 50% of all insured individuals annually [46]. As described previously, the Ministry of Justice pays DAA therapy costs for individuals in detention. Asylum seekers can be considered for HCV treatment, but only when a residence permit is granted.

**Fig. 2:**
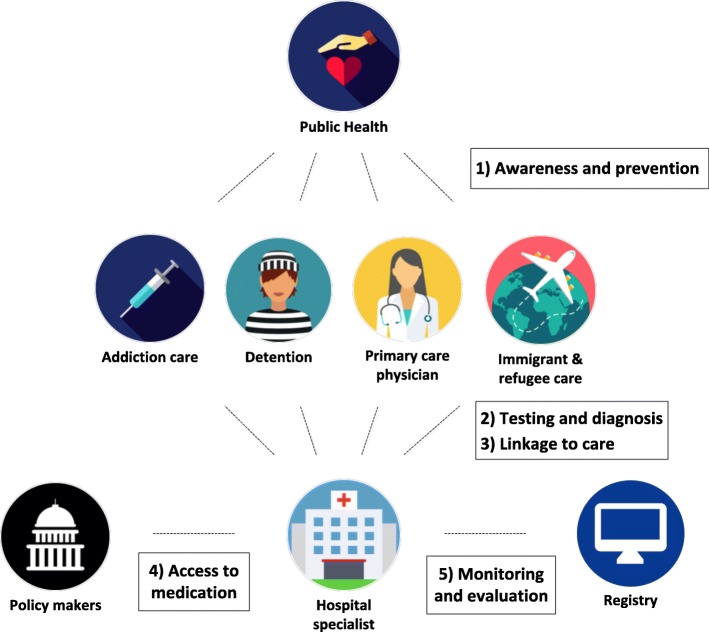
Dutch HCV healthcare stakeholders

**Fig. 3:**
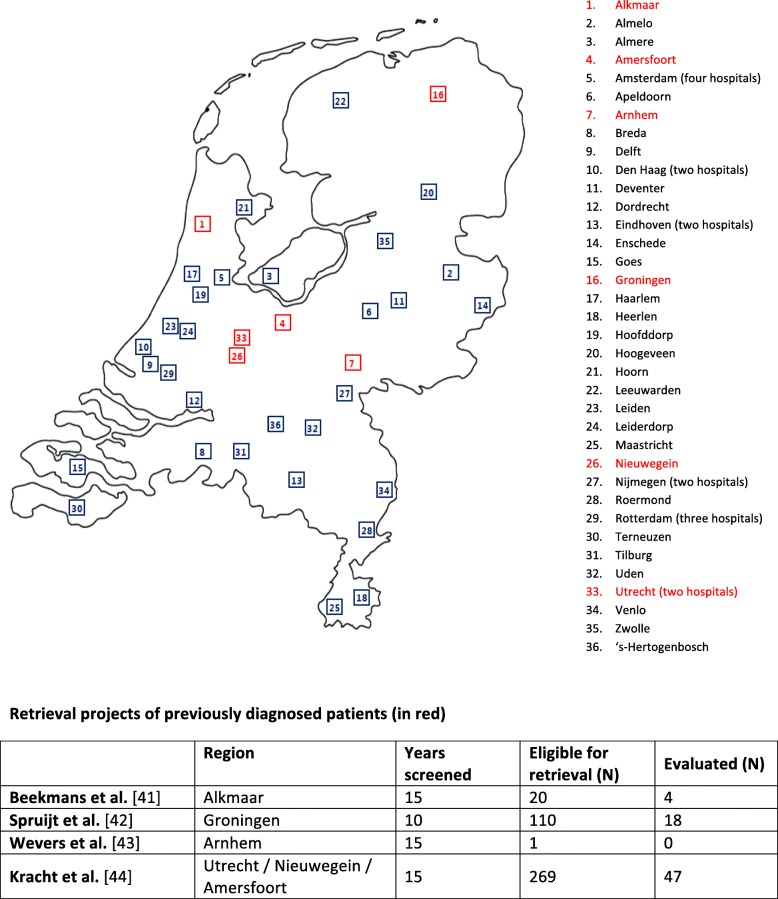
All 45 hepatitis treatment centers in the Netherlands [41–44]

Although a wide variety of stakeholders is involved in the detection of and care for patients with a chronic HCV infection in the Netherlands (Fig. 2), until now treatment of HCV remains under the responsibility of hospital-based-physicians (specialists in hepatology or infectious diseases physicians working in designated hepatitis centers). There are 45 designated hepatitis centers in the Netherlands (Fig. 3) which can all be attended to on referral by the PCP. Physicians in all hepatitis treatment centers can consult the Dutch national “guidebook” on HCV treatment (www.HCVrichtsnoer.nl) that summarizes the recommendations of the main international guidelines on HCV treatment. The ‘HCV richtsnoer’ provides guidance for the appropriate DAA regimen selection with an update being published after each major international guideline revision [47].

### Monitoring and evaluation

Adequate monitoring and registration of all patients in each step of the HCV healthcare cascade is essential to achieve HCV elimination. When drop out at any of the stages of the HCV healthcare cascade has been reduced to zero, elimination will have been achieved. By the Dutch public health act, in 1999 it became mandatory to report acute HCV infections to the local Public Health Services and hence incident infections are recorded since then. Until now, chronic HCV infections do not require notification but this is about to change in 2018. In addition, a pilot project was initiated in 2017 by the Dutch Association of Internal Medicine (NIV) and Gastroenterology and Hepatology (NVMDL) specialists with 8 participating hospitals aiming to create a registry for treatment uptake and outcome of all viral hepatitis B & C mono-infections. This project was able to benefit from the existing registration system of the HIV Monitoring Foundation (SHM) that has already been used to monitor both HIV-mono and HIV/hepatitis B or C co-infected patients for decades [48]. The previously mentioned national retrieval project CELINE aspires to further, both retrospectively and prospectively, complete this registry in the upcoming years.

The Dutch Central Bureau of Statistics (CBS) collects data on the primary cause of death in the general population and based on this information, the annual viral hepatitis C and B related mortality was estimated at +/− 500 deaths per year between 2002 and 2015 [31]. The viral hepatitis related mortality has not yet decreased as a consequence of the introduction of DAA-therapy. The van Creveldkliniek hemophilia treatment center reported a mortality rate of 28% in patients with hemophilia and chronic HCV of which 28% was liver-related (median follow-up of 31 years since HCV infection) [25]. These numbers however date from the pre-DAA era. The SHM registers the cause of death in HIV mono- and HIV/HCV coinfected however viral hepatitis related mortality in HIV/HCV coinfected individuals has not been explicitly described [24].

## Future direction for hepatitis C (micro-) elimination in the Netherlands

The present Dutch situation with low HCV seroprevalence (0.1–0.4%) [7, 11] and a limited number of new infections [17, 23] is an excellent starting point for final HCV elimination. Some experts argue that the proportion of undiagnosed or ‘hidden’ HCV patients may actually be smaller than previously estimated as exemplified by a large high prevalence birth cohort screening project in the South of the Netherlands (*n* = 3434 patients) that did not identify any active HCV infections [49]. A study modelling the future HCV burden of disease in the Netherlands estimated an 85% reduction in chronic HCV infections by the year 2030, if treatment rate can be scaled up adequately [50]. In the past years, numerous Dutch micro-elimination projects have consequently put their efforts into enhancing the screening, linkage to care and finally the HCV treatment uptake in various risk group populations [27, 30, 32–35, 37, 38, 41–43]. Taking all endeavors into account, a drastic future reduction in the Dutch pool of chronic HCV-infected can be envisioned.

Important caveats however do remain in various stages of the Dutch HCV continuum of care which will have to be addressed before HCV micro-elimination can be achieved. First of all, the proportion of chronic HCV patients who has been lost to follow-up is substantial [41–43]. The nationwide roll out of the pilot project for retrieval but also registration of viral hepatitis C mono-infections (CELINE) therefore constitutes an important challenge to complete in the near future [45]. Secondly, as can be observed in the micro-elimination table, data on exact prevalence and therapy uptake is still unknown for many target groups (Table 1). The progress against HCV can thus not be tracked appropriately. When successfully executed, CELINE will substantially contribute to overcoming this issue by entering the data of > 95% of previously diagnosed HCV patients who have been retrieved in a central registry. Thirdly, despite the existence of various professional HCV screening guidelines, current screening strategies do not adequately target several high prevalence risk groups and a considerable group might still be unware of their disease. Specific improvements have to be made in the identification and linkage to care of HCV patients in addiction care centers, those originating from high endemic HCV countries immigrating to the Netherlands and also the general population as they constitute the major contributors to the Dutch HCV burden of disease (although overestimation of the HCV prevalence cannot be ruled out) [7]. Also, HIV-negative MSM who engage in high-risk sexual behavior (e.g. those receiving HIV Pre-Exposure Prophylaxis or PrEP) recently emerged as another subgroup with a relatively high HCV incidence (0.7–1.3 per 100 person-years) [51–53]. In the SHM HIV/HCV coinfected cohort, high DAA-therapy uptake of 76% has led to a decrease in HCV incident infections by half (from 11.2 to 5.5 per 1000 person-years) but a substantial (re-) infection rate remains [23]. Modeling studies describe that behavioral counseling in addition to treatment scale-up may be effective in further reducing the long-term HCV prevalence [54, 55].

With the current diminishing prevalence, the upcoming challenge will be to improve case finding strategies in order to accomplish higher treatment rates and to avoid a ‘diagnostic burn-out’ (i.e. the amount of treatments is reduced to zero because patients remain unware of their disease and therefore will not receive therapy) [56]. This diagnostic endeavor is preferably taken on by the PCPs as they encounter the majority of the Dutch population at risk for HCV in their practices. Since knowledge of HCV in Dutch PCPs is deemed insufficient, focus should shift towards educating PCPs about viral hepatitis C. With the current simplified DAA therapy regimens, treatment of patients with none to mild fibrosis may even be transferred to the PCPs in the future.

## Conclusion

The current Dutch HCV epidemiology with both low prevalence and incidence in combination with universal access to DAA therapy, favors a future HCV elimination scenario. The micro-elimination method, that delivers targeted interventions at pre-defined HCV risk groups, is frequently applied to improve the Dutch HCV healthcare cascade and constitutes a pragmatic and efficient approach. To avoid an eventual diagnostic burn-out, efforts towards case-finding should be intensified. Although challenges remain, the Netherlands continues to be one of the global frontrunners in its efforts to national viral hepatitis C elimination. Whether this goal may actually be achieved by the year 2030, remains to be elucidated in the next couple of years.

## Abbreviations

DAA: Direct-acting antiviral
ELPA: European liver patients association
GIP: Drug information system of the national health care institute
HCV: Hepatitis C virus
HIV: Human immunodeficiency virus
NHG: Dutch college of general practitioners
NIV: Dutch association of internal medicine
NVMDL: Dutch association of gastroenterology and hepatology
PCP: Primary care physicians
PWID: People who (ever) inject(ed) drugs
SHM: Dutch HIV monitoring foundation
WHO: World health organization
